# (2*E*)-3-(3-Benzyl­oxyphen­yl)-1-(2-hydroxy-5-methyl­phen­yl)prop-2-en-1-one

**DOI:** 10.1107/S1600536811016977

**Published:** 2011-05-11

**Authors:** Hoong-Kun Fun, Suhana Arshad, B. K. Sarojini, V. Musthafa Khaleel, B. Narayana

**Affiliations:** a X-ray Crystallography Unit, School of Physics, Universiti Sains Malaysia, 11800 USM, Penang, Malaysia; b Department of Chemistry, P. A. College of Engineering, Mangalore 574 153, India; c Department of Studies in Chemistry, Mangalore University, Mangalagangotri, Mangalore 574 199, India

## Abstract

In the mol­ecule of the title compound, C_23_H_20_O_3_, an intra­molecular O—H⋯O hydrogen bond generates an *S*(6) ring. The central benzene ring makes dihedral angles of 80.17 (8) and 16.99 (7)°, respectively, with the benz­yloxy and hy­droxy­methyl phenyl rings. In the crystal, mol­ecules are linked *via* inter­molecular C—H⋯O hydrogen bonds to form dimers. The dimers are connected by C—H⋯O hydrogen bonds and C—H⋯π inter­actions to form columns down the *b* axis.

## Related literature

For general background and applications of chalcones, see: Awad *et al.* (1960 ▶); Coudert *et al.* (1988 ▶); Insuasty *et al.* (1992 ▶, 1997 ▶); Kolos *et al.* (1996 ▶); Sarojini *et al.* (2006 ▶); Shettigar *et al.* (2010 ▶); Samshuddin *et al.* (2010 ▶); Fun *et al.* (2010 ▶). For related structures, see: Butcher *et al.* (2006 ▶); Ravishankar *et al.* (2003 ▶, 2005 ▶); Narayana *et al.* (2007 ▶); Sarojini, Narayana *et al.* (2007 ▶); Sarojini, Yathirajan *et al.* (2007 ▶); Sharma *et al.* (1997 ▶); Jasinski *et al.* (2011 ▶). For hydrogen-bond motifs, see: Bernstein *et al.* (1995 ▶). For bond-length data, see: Allen *et al.* (1987 ▶).
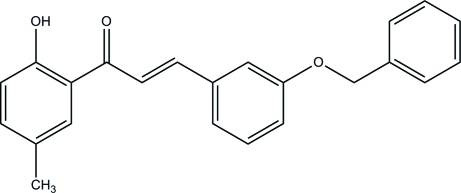


## Experimental

### 

#### Crystal data

C_23_H_20_O_3_
*M* *_r_* = 344.39
Triclinic, 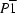

*a* = 8.7308 (5) Å
*b* = 9.5721 (5) Å
*c* = 11.5286 (6) Å
α = 106.547 (1)°
β = 94.572 (1)°
γ = 101.671 (1)°
*V* = 894.74 (8) Å^3^
*Z* = 2
Mo *K*α radiation
μ = 0.08 mm^−1^
*T* = 296 K
0.42 × 0.37 × 0.28 mm

#### Data collection

Bruker SMART APEXII DUO CCD area-detector diffractometer
Absorption correction: multi-scan (*SADABS*; Bruker, 2009 ▶) *T*_min_ = 0.966, *T*_max_ = 0.977
18270 measured reflections
5238 independent reflections
3853 reflections with *I* > 2σ(*I*)
*R*_int_ = 0.020

#### Refinement

*R*[*F* ^2^ > 2σ(*F* ^2^)] = 0.049
*wR*(*F* ^2^) = 0.163
*S* = 1.03
5238 reflections
240 parameters
H atoms treated by a mixture of independent and constrained refinement
Δρ_max_ = 0.26 e Å^−3^
Δρ_min_ = −0.23 e Å^−3^

### 

Data collection: *APEX2* (Bruker, 2009 ▶); cell refinement: *SAINT* (Bruker, 2009 ▶); data reduction: *SAINT*; program(s) used to solve structure: *SHELXTL* (Sheldrick, 2008 ▶); program(s) used to refine structure: *SHELXTL*; molecular graphics: *SHELXTL*; software used to prepare material for publication: *SHELXTL* and *PLATON* (Spek, 2009 ▶).

## Supplementary Material

Crystal structure: contains datablocks global, I. DOI: 10.1107/S1600536811016977/ci5187sup1.cif

Structure factors: contains datablocks I. DOI: 10.1107/S1600536811016977/ci5187Isup2.hkl

Supplementary material file. DOI: 10.1107/S1600536811016977/ci5187Isup3.cml

Additional supplementary materials:  crystallographic information; 3D view; checkCIF report

## Figures and Tables

**Table 1:** Hydrogen-bond geometry (Å, °) *Cg*1 is the centroid of the C17–C22 ring.

*D*—H⋯*A*	*D*—H	H⋯*A*	*D*⋯*A*	*D*—H⋯*A*
O2—H1*O*2⋯O3	0.93 (2)	1.65 (3)	2.521 (2)	155 (3)
C16—H16*B*⋯O3^i^	0.97	2.60	3.445 (2)	146
C22—H22*A*⋯O2^ii^	0.93	2.56	3.435 (2)	158
C11—H11*A*⋯*Cg*1^iii^	0.93	2.80	3.660 (2)	153

Symmetry codes: (i) 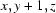; (ii) 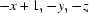; (iii) 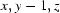.

**Fig. 1.**
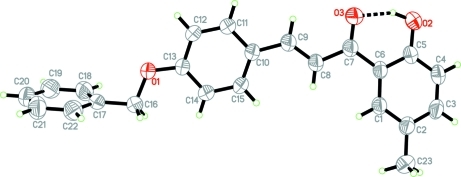
The molecular structure of the title compound, showing 50% probability displacement ellipsoids and the atom-numbering scheme. The dashed line indicates an intramolecular hydrogen bond.

**Fig. 2.**
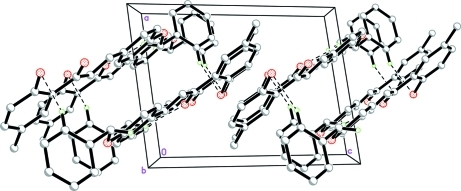
The crystal packing of the title compound. Dashed lines represent hydrogen bonds.

## supplementary crystallographic information

### Comment

Chalcones (1,3-diarylpropenones) have been widely used as starting materials in numerous synthetic reactions (Awad *et al.*, 1960; Coudert *et al.*, 1988) including the preparation of fused-ring heterocyclic compounds (Insuasty *et al.*, 1992, 1997; Kolos *et al.*, 1996; Samshuddin *et al.*, 2010; Fun *et al.*, 2010). Chalcones are also finding application as organic nonlinear optical materials (NLO) for their SHG conversion efficiency (Sarojini *et al.*, 2006; Shettigar *et al.*, 2010). The crystal structures of some of the related chalcones *viz* 1-(3,4-dimethoxyphenyl)-3-(3-methylphenyl)prop-2-en-1-one (Sharma *et al.*, 1997), 3-(3,4-dimethoxyphenyl)-1-(4-hydroxy-phenyl)prop-2-en-1-one (Ravishankar *et al.*, 2003), 1-(4-chlorophenyl)-3-(4-hydroxyphenyl) prop-2-en-1-one (Ravishankar *et al.*, 2005), 3-(3,4-dimethoxyphenyl)-1- (4-fluorophenyl)prop-2-en-1-one (Butcher *et al.*, 2006), 3-(2-chlorophenyl)-1-(4-hydroxyphenyl)prop-2-en-1-one (Narayana *et al.*, 2007), (2*E*)-1-(2-hydroxyphenyl)-3-(4-methoxy-phenyl)prop-2-en-1-one, (2*E*)-1-(2-hydroxyphenyl)-3-[4-(methyl-sulfanyl)phenyl]prop-2-en-1-one (Sarojini, Narayana *et al.*, 2007; Sarojini, Yathirajan *et al.*, 2007) and (2*E*)-3- (3,4-dimethoxyphenyl)-1-(4-hydroxyphenyl)prop-2-en-1-one (Jasinski *et al.*, 2011) have been reported. In continuation to our studies on structures of chalcones, we report here the crystal structure of a new chalcone, the title compound.

In the molecular structure (Fig. 1), an intramolecular O2—H1O2···O3 hydrogen bond (Table 1) forming an *S*(6) ring motif (Bernstein *et al.*, 1995) is observed. The C1–C6 and C10–C15 benzene rings form a dihedral angle of 16.99 (7)° between them. In addition, they also make dihedral angles of 69.01 (7) and 80.17 (8)°, respectively, with the terminal phenyl ring (C17–C22). Bond lengths (Allen *et al.*, 1987) and angles are within normal range.

The crystal packing is shown in Fig. 2. The molecules are linked by intermolecular C22—H22A···O2 hydrogen bonds (Table 1) to form dimers. Furthermore, these dimers are connected by intermolecular C16—H16B···O3 hydrogen bonds (Table 1) to form columns down the *b* axis. The C—H···π interactions (Table 1) which involve C11 and the C17–C22 phenyl ring further stabilize the crystal structure.

### Experimental

2-Hydroxy-5-methoxyacetophenone (1.66 g, 0.01 mol) was mixed with 4-benzyloxybenzaldehyde (2.12 g, 0.01 mol) and dissolved in ethanol (30 ml). To this solution, 3 ml of KOH (50%, 10 mL) was added at 5°C. The reaction mixture was stirred for 5 h and poured on to crushed ice. The pH of this mixture was adjusted to 3–4 with 2 *M* HCl aqueous solution. The resulting crude yellow solid was filtered, washed successively with dilute HCl solution and distilled water and finally recrystallized from ethanol (95%) to give the pure chalcone. Crystals suitable for X-ray diffraction studies were grown by slow evaporation of the solution of the compound in ethyl alcohol-DMF (4:1) mixture (*m.p.* 393–397 K). Composition: found (calculated) for C_23_H_20_O_3_: C 76.65 (76.63), H 5.59 (5.57).

### Refinement

Atom H1O2  was located in a difference map and refined freely [O–H = 0.93 (3) Å]. The remaining H atoms were positioned geometrically [C–H = 0.93 or 0.97 Å] and refined using a riding model with *U*_iso_(H) = 1.2 or 1.5 *U*_eq_(C). A rotating group model was applied to the methyl group.

### Crystal data

**Table:**

C_23_H_20_O_3_	*Z* = 2
*M**_r_* = 344.39	*F*(000) = 364
Triclinic, *P*1	*D*_x_ = 1.278 Mg m^−^^3^
Hall symbol: -P 1	Mo *K*α radiation, λ = 0.71073 Å
*a* = 8.7308 (5) Å	Cell parameters from 5803 reflections
*b* = 9.5721 (5) Å	θ = 2.8–30.0°
*c* = 11.5286 (6) Å	µ = 0.08 mm^−^^1^
α = 106.547 (1)°	*T* = 296 K
β = 94.572 (1)°	Block, orange
γ = 101.671 (1)°	0.42 × 0.37 × 0.28 mm
*V* = 894.74 (8) Å^3^	

### Data collection

**Table:**

Bruker SMART APEXII DUO CCD area-detector diffractometer	5238 independent reflections
Radiation source: fine-focus sealed tube	3853 reflections with *I* > 2σ(*I*)
graphite	*R*_int_ = 0.020
φ and ω scans	θ_max_ = 30.1°, θ_min_ = 2.3°
Absorption correction: multi-scan (*SADABS*; Bruker, 2009)	*h* = −12→12
*T*_min_ = 0.966, *T*_max_ = 0.977	*k* = −13→13
18270 measured reflections	*l* = −15→16

### Refinement

**Table:**

Refinement on *F*^2^	Primary atom site location: structure-invariant direct methods
Least-squares matrix: full	Secondary atom site location: difference Fourier map
*R*[*F*^2^ > 2σ(*F*^2^)] = 0.049	Hydrogen site location: inferred from neighbouring sites
*wR*(*F*^2^) = 0.163	H atoms treated by a mixture of independent and constrained refinement
*S* = 1.03	*w* = 1/[σ^2^(*F*_o_^2^) + (0.0847*P*)^2^ + 0.1176*P*] where *P* = (*F*_o_^2^ + 2*F*_c_^2^)/3
5238 reflections	(Δ/σ)_max_ = 0.001
240 parameters	Δρ_max_ = 0.26 e Å^−^^3^
0 restraints	Δρ_min_ = −0.23 e Å^−^^3^

### Special details

**Table:**

Geometry. All e.s.d.'s (except the e.s.d. in the dihedral angle between two l.s. planes) are estimated using the full covariance matrix. The cell e.s.d.'s are taken into account individually in the estimation of e.s.d.'s in distances, angles and torsion angles; correlations between e.s.d.'s in cell parameters are only used when they are defined by crystal symmetry. An approximate (isotropic) treatment of cell e.s.d.'s is used for estimating e.s.d.'s involving l.s. planes.
Refinement. Refinement of *F*^2^ against ALL reflections. The weighted *R*-factor *wR* and goodness of fit *S* are based on *F*^2^, conventional *R*-factors *R* are based on *F*, with *F* set to zero for negative *F*^2^. The threshold expression of *F*^2^ > σ(*F*^2^) is used only for calculating *R*-factors(gt) *etc*. and is not relevant to the choice of reflections for refinement. *R*-factors based on *F*^2^ are statistically about twice as large as those based on *F*, and *R*- factors based on ALL data will be even larger.

### Fractional atomic coordinates and isotropic or equivalent isotropic displacement parameters (Å^2^)

**Table:**

	*x*	*y*	*z*	*U*_iso_*/*U*_eq_	
O1	0.16772 (13)	0.30000 (11)	−0.13861 (10)	0.0679 (3)	
O2	0.43323 (13)	−0.41373 (11)	0.37191 (11)	0.0644 (3)	
O3	0.37319 (15)	−0.32210 (11)	0.19276 (10)	0.0681 (3)	
C1	0.66751 (15)	−0.03177 (14)	0.42278 (11)	0.0459 (3)	
H1A	0.6795	0.0439	0.3861	0.055*	
C2	0.76064 (15)	−0.00671 (14)	0.53313 (11)	0.0485 (3)	
C3	0.73915 (17)	−0.12245 (17)	0.58579 (13)	0.0552 (3)	
H3A	0.8003	−0.1083	0.6599	0.066*	
C4	0.63119 (17)	−0.25623 (16)	0.53216 (13)	0.0561 (3)	
H4A	0.6195	−0.3308	0.5700	0.067*	
C5	0.53927 (15)	−0.28026 (14)	0.42114 (12)	0.0475 (3)	
C6	0.55590 (14)	−0.16674 (13)	0.36425 (10)	0.0428 (2)	
C7	0.45402 (16)	−0.19356 (14)	0.24783 (11)	0.0475 (3)	
C8	0.44225 (16)	−0.07006 (14)	0.19893 (11)	0.0484 (3)	
H8A	0.5083	0.0245	0.2364	0.058*	
C9	0.33780 (17)	−0.09225 (15)	0.10131 (12)	0.0526 (3)	
H9A	0.2805	−0.1906	0.0646	0.063*	
C10	0.30100 (16)	0.01655 (14)	0.04420 (11)	0.0490 (3)	
C11	0.1932 (2)	−0.03475 (16)	−0.06343 (15)	0.0703 (5)	
H11A	0.1479	−0.1369	−0.0963	0.084*	
C12	0.1525 (2)	0.06119 (17)	−0.12187 (15)	0.0732 (5)	
H12A	0.0811	0.0237	−0.1939	0.088*	
C13	0.21741 (16)	0.21433 (14)	−0.07401 (12)	0.0511 (3)	
C14	0.32657 (17)	0.26857 (15)	0.03205 (12)	0.0536 (3)	
H14A	0.3720	0.3708	0.0644	0.064*	
C15	0.36739 (17)	0.16958 (15)	0.08932 (12)	0.0537 (3)	
H15A	0.4413	0.2066	0.1600	0.064*	
C16	0.24447 (19)	0.45417 (15)	−0.10512 (15)	0.0644 (4)	
H16A	0.3565	0.4657	−0.1101	0.077*	
H16B	0.2321	0.5047	−0.0218	0.077*	
C17	0.17020 (16)	0.52033 (13)	−0.19193 (13)	0.0526 (3)	
C18	0.01997 (18)	0.54490 (19)	−0.18521 (17)	0.0677 (4)	
H18A	−0.0364	0.5200	−0.1259	0.081*	
C19	−0.04746 (19)	0.60627 (19)	−0.26593 (18)	0.0711 (4)	
H19A	−0.1485	0.6225	−0.2604	0.085*	
C20	0.0337 (2)	0.64284 (18)	−0.35342 (15)	0.0685 (4)	
H20A	−0.0119	0.6837	−0.4077	0.082*	
C21	0.1825 (2)	0.6194 (2)	−0.36131 (15)	0.0724 (4)	
H21A	0.2380	0.6446	−0.4209	0.087*	
C22	0.25088 (19)	0.55839 (17)	−0.28101 (14)	0.0615 (4)	
H22A	0.3521	0.5429	−0.2871	0.074*	
C23	0.87881 (19)	0.13954 (18)	0.59510 (15)	0.0658 (4)	
H23A	0.8420	0.2194	0.5759	0.099*	
H23B	0.9787	0.1346	0.5670	0.099*	
H23C	0.8911	0.1579	0.6820	0.099*	
H1O2	0.392 (3)	−0.406 (3)	0.298 (2)	0.110 (8)*	

### Atomic displacement parameters (Å^2^)

**Table:**

	*U*^11^	*U*^22^	*U*^33^	*U*^12^	*U*^13^	*U*^23^
O1	0.0792 (7)	0.0450 (5)	0.0711 (7)	0.0010 (5)	−0.0301 (5)	0.0254 (5)
O2	0.0715 (6)	0.0486 (5)	0.0734 (7)	0.0049 (4)	−0.0062 (5)	0.0304 (5)
O3	0.0917 (8)	0.0466 (5)	0.0566 (6)	0.0033 (5)	−0.0159 (5)	0.0173 (4)
C1	0.0530 (6)	0.0458 (6)	0.0430 (6)	0.0145 (5)	0.0042 (5)	0.0188 (5)
C2	0.0505 (6)	0.0519 (6)	0.0451 (6)	0.0164 (5)	0.0027 (5)	0.0162 (5)
C3	0.0606 (7)	0.0635 (8)	0.0473 (6)	0.0225 (6)	−0.0012 (5)	0.0232 (6)
C4	0.0668 (8)	0.0557 (7)	0.0561 (7)	0.0199 (6)	0.0032 (6)	0.0310 (6)
C5	0.0523 (6)	0.0452 (6)	0.0514 (6)	0.0162 (5)	0.0064 (5)	0.0217 (5)
C6	0.0498 (6)	0.0437 (6)	0.0397 (5)	0.0165 (5)	0.0050 (4)	0.0168 (4)
C7	0.0571 (7)	0.0454 (6)	0.0417 (6)	0.0137 (5)	0.0029 (5)	0.0162 (5)
C8	0.0589 (7)	0.0453 (6)	0.0431 (6)	0.0135 (5)	0.0012 (5)	0.0177 (5)
C9	0.0681 (8)	0.0453 (6)	0.0431 (6)	0.0117 (5)	−0.0031 (5)	0.0156 (5)
C10	0.0585 (7)	0.0458 (6)	0.0415 (6)	0.0108 (5)	−0.0038 (5)	0.0157 (5)
C11	0.0894 (11)	0.0432 (7)	0.0645 (9)	−0.0001 (7)	−0.0308 (8)	0.0175 (6)
C12	0.0889 (11)	0.0487 (7)	0.0666 (9)	−0.0018 (7)	−0.0384 (8)	0.0198 (7)
C13	0.0563 (7)	0.0444 (6)	0.0507 (7)	0.0082 (5)	−0.0092 (5)	0.0190 (5)
C14	0.0607 (7)	0.0423 (6)	0.0500 (7)	0.0045 (5)	−0.0112 (5)	0.0128 (5)
C15	0.0617 (7)	0.0495 (6)	0.0436 (6)	0.0075 (5)	−0.0129 (5)	0.0139 (5)
C16	0.0729 (9)	0.0446 (7)	0.0673 (9)	0.0019 (6)	−0.0206 (7)	0.0211 (6)
C17	0.0583 (7)	0.0372 (5)	0.0567 (7)	0.0047 (5)	−0.0095 (6)	0.0153 (5)
C18	0.0572 (8)	0.0691 (9)	0.0813 (10)	0.0046 (7)	0.0034 (7)	0.0390 (8)
C19	0.0524 (7)	0.0668 (9)	0.0942 (12)	0.0110 (7)	−0.0089 (7)	0.0323 (9)
C20	0.0812 (10)	0.0570 (8)	0.0655 (9)	0.0160 (7)	−0.0137 (8)	0.0229 (7)
C21	0.0939 (12)	0.0728 (10)	0.0581 (9)	0.0244 (9)	0.0113 (8)	0.0287 (8)
C22	0.0650 (8)	0.0560 (8)	0.0647 (8)	0.0207 (6)	0.0057 (7)	0.0172 (7)
C23	0.0657 (9)	0.0635 (8)	0.0607 (8)	0.0067 (7)	−0.0080 (7)	0.0178 (7)

### Geometric parameters (Å, °)

**Table:**

O1—C13	1.3622 (14)	C11—H11A	0.93
O1—C16	1.4178 (16)	C12—C13	1.3884 (18)
O2—C5	1.3551 (16)	C12—H12A	0.93
O2—H1O2	0.93 (3)	C13—C14	1.3876 (17)
O3—C7	1.2439 (16)	C14—C15	1.3843 (18)
C1—C2	1.3854 (17)	C14—H14A	0.93
C1—C6	1.4011 (17)	C15—H15A	0.93
C1—H1A	0.93	C16—C17	1.4990 (19)
C2—C3	1.3958 (19)	C16—H16A	0.97
C2—C23	1.504 (2)	C16—H16B	0.97
C3—C4	1.371 (2)	C17—C22	1.381 (2)
C3—H3A	0.93	C17—C18	1.383 (2)
C4—C5	1.3889 (18)	C18—C19	1.386 (2)
C4—H4A	0.93	C18—H18A	0.93
C5—C6	1.4104 (16)	C19—C20	1.363 (3)
C6—C7	1.4764 (16)	C19—H19A	0.93
C7—C8	1.4647 (17)	C20—C21	1.368 (3)
C8—C9	1.3286 (17)	C20—H20A	0.93
C8—H8A	0.93	C21—C22	1.384 (2)
C9—C10	1.4538 (17)	C21—H21A	0.93
C9—H9A	0.93	C22—H22A	0.93
C10—C15	1.3880 (18)	C23—H23A	0.96
C10—C11	1.3950 (18)	C23—H23B	0.96
C11—C12	1.3674 (19)	C23—H23C	0.96
			
C13—O1—C16	118.66 (10)	O1—C13—C12	115.52 (11)
C5—O2—H1O2	102.3 (15)	C14—C13—C12	119.36 (12)
C2—C1—C6	122.54 (11)	C15—C14—C13	119.56 (12)
C2—C1—H1A	118.7	C15—C14—H14A	120.2
C6—C1—H1A	118.7	C13—C14—H14A	120.2
C1—C2—C3	117.23 (12)	C14—C15—C10	121.81 (11)
C1—C2—C23	121.74 (12)	C14—C15—H15A	119.1
C3—C2—C23	121.03 (12)	C10—C15—H15A	119.1
C4—C3—C2	122.34 (12)	O1—C16—C17	107.69 (11)
C4—C3—H3A	118.8	O1—C16—H16A	110.2
C2—C3—H3A	118.8	C17—C16—H16A	110.2
C3—C4—C5	119.85 (12)	O1—C16—H16B	110.2
C3—C4—H4A	120.1	C17—C16—H16B	110.2
C5—C4—H4A	120.1	H16A—C16—H16B	108.5
O2—C5—C4	117.85 (11)	C22—C17—C18	118.30 (13)
O2—C5—C6	122.05 (11)	C22—C17—C16	120.52 (14)
C4—C5—C6	120.09 (12)	C18—C17—C16	121.18 (14)
C1—C6—C5	117.95 (11)	C17—C18—C19	120.67 (15)
C1—C6—C7	122.97 (10)	C17—C18—H18A	119.7
C5—C6—C7	119.07 (11)	C19—C18—H18A	119.7
O3—C7—C8	119.66 (11)	C20—C19—C18	120.23 (15)
O3—C7—C6	119.36 (11)	C20—C19—H19A	119.9
C8—C7—C6	120.94 (11)	C18—C19—H19A	119.9
C9—C8—C7	120.42 (12)	C19—C20—C21	119.87 (14)
C9—C8—H8A	119.8	C19—C20—H20A	120.1
C7—C8—H8A	119.8	C21—C20—H20A	120.1
C8—C9—C10	128.73 (12)	C20—C21—C22	120.30 (16)
C8—C9—H9A	115.6	C20—C21—H21A	119.8
C10—C9—H9A	115.6	C22—C21—H21A	119.8
C15—C10—C11	117.27 (12)	C17—C22—C21	120.62 (15)
C15—C10—C9	124.19 (11)	C17—C22—H22A	119.7
C11—C10—C9	118.54 (12)	C21—C22—H22A	119.7
C12—C11—C10	121.74 (13)	C2—C23—H23A	109.5
C12—C11—H11A	119.1	C2—C23—H23B	109.5
C10—C11—H11A	119.1	H23A—C23—H23B	109.5
C11—C12—C13	120.24 (12)	C2—C23—H23C	109.5
C11—C12—H12A	119.9	H23A—C23—H23C	109.5
C13—C12—H12A	119.9	H23B—C23—H23C	109.5
O1—C13—C14	125.12 (11)		
			
C6—C1—C2—C3	−0.22 (19)	C9—C10—C11—C12	179.73 (17)
C6—C1—C2—C23	−179.44 (13)	C10—C11—C12—C13	−0.6 (3)
C1—C2—C3—C4	0.0 (2)	C16—O1—C13—C14	7.7 (2)
C23—C2—C3—C4	179.26 (14)	C16—O1—C13—C12	−171.44 (16)
C2—C3—C4—C5	0.4 (2)	C11—C12—C13—O1	−179.34 (17)
C3—C4—C5—O2	−179.51 (12)	C11—C12—C13—C14	1.5 (3)
C3—C4—C5—C6	−0.6 (2)	O1—C13—C14—C15	−179.99 (14)
C2—C1—C6—C5	0.00 (18)	C12—C13—C14—C15	−0.9 (2)
C2—C1—C6—C7	178.84 (11)	C13—C14—C15—C10	−0.6 (2)
O2—C5—C6—C1	179.27 (11)	C11—C10—C15—C14	1.4 (2)
C4—C5—C6—C1	0.41 (19)	C9—C10—C15—C14	−179.16 (13)
O2—C5—C6—C7	0.39 (19)	C13—O1—C16—C17	178.62 (13)
C4—C5—C6—C7	−178.47 (11)	O1—C16—C17—C22	−107.22 (16)
C1—C6—C7—O3	170.34 (13)	O1—C16—C17—C18	72.78 (18)
C5—C6—C7—O3	−10.84 (19)	C22—C17—C18—C19	0.0 (2)
C1—C6—C7—C8	−12.05 (19)	C16—C17—C18—C19	179.95 (14)
C5—C6—C7—C8	166.77 (11)	C17—C18—C19—C20	0.2 (3)
O3—C7—C8—C9	4.9 (2)	C18—C19—C20—C21	−0.2 (3)
C6—C7—C8—C9	−172.75 (12)	C19—C20—C21—C22	0.1 (3)
C7—C8—C9—C10	175.62 (13)	C18—C17—C22—C21	0.0 (2)
C8—C9—C10—C15	−3.7 (2)	C16—C17—C22—C21	179.96 (14)
C8—C9—C10—C11	175.79 (16)	C20—C21—C22—C17	0.0 (2)
C15—C10—C11—C12	−0.8 (3)		

### Hydrogen-bond geometry (Å, °)

**Table:**

*D*—H···*A*	*D*—H	H···*A*	*D*···*A*	*D*—H···*A*
O2—H1O2···O3	0.93 (2)	1.65 (3)	2.521 (2)	155 (3)
C16—H16B···O3^i^	0.97	2.60	3.445 (2)	146
C22—H22A···O2^ii^	0.93	2.56	3.435 (2)	158
C11—H11A···Cg1^iii^	0.93	2.80	3.660 (2)	153

Symmetry codes: (i) *x*, *y*+1, *z*; (ii) −*x*+1, −*y*, −*z*; (iii) *x*, *y*−1, *z*.
